# Supplementary material for: Fabrication of Celecoxib PVP Microparticles Stabilized by Gelucire 48/16 via Electrospraying for Enhanced Anti-Inflammatory Action
Source: Pharmaceuticals (Basel). 2023 Feb 8;16(2):258. doi: 10.3390/ph16020258 (PMC9960083; doi:10.3390/ph16020258)
Supplement: Supplementary file 1 [file pharmaceuticals-16-00258-s001.zip › pharmaceuticals-2158894-supplementary.pdf]

Inflammation-induced by carrageenan

It was performed as previously described Using a subcutaneous (SC) injection of 0.2 ml of carrageenan solution (1%w/v dissolved in sterile saline), the inflammation was induced in the sub-planter right hind paw. We used the left hind paw as a control as it wasn't injected with carrageenan.

Table S1. The sequences of the utilized primers.

Gene	Sequence
Beta actin	Forward 5'-GTCAGGTCATCACTATCGGCAAT-3'<br>Reverse 5'- AGAGGTCTTTACGGATGTCAACGT-3'
IL-1β	Forward 5'-CACCTCTCAAGCAGAGCACAG-3'<br>Reverse 5'-GGGTTCCATGGTGAAGTCAAC-3'
IL-6	Forward 5'-GCCCTTCAGGAACAGCTATGA-3'<br>Reverse 5'-TGTCAACAACATCAGTCCCAAGA-3'

Z-Average (d.nm):	589.8
PdI:	0.283
Intercept:	0.896
Result quality :	Good

	Size (d.nm):	% Intensity:	St Dev (d.n...
Peak 1:	521.5	100.0	124.7
Peak 2:	0.000	0.0	0.000
Peak 3:	0.000	0.0	0.000

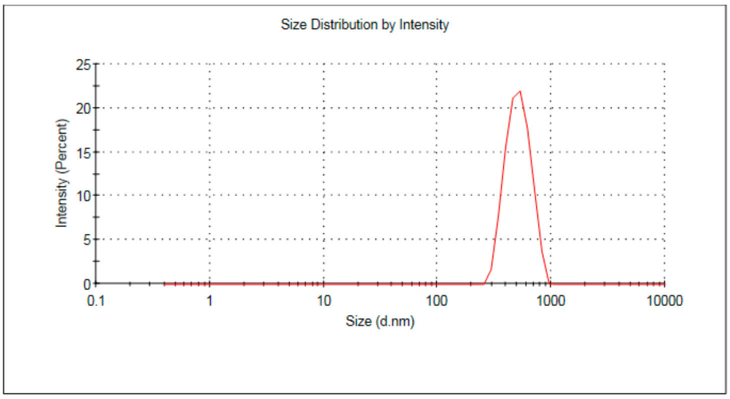

Z-Average (d.nm):	573.9
PdI:	0.246
Intercept:	0.891
Result quality :	Good

	Size (d.nm):	% Intensity:	St Dev (d.n...
Peak 1:	527.1	98.6	132.3
Peak 2:	5494	1.4	213.4
Peak 3:	0.000	0.0	0.000

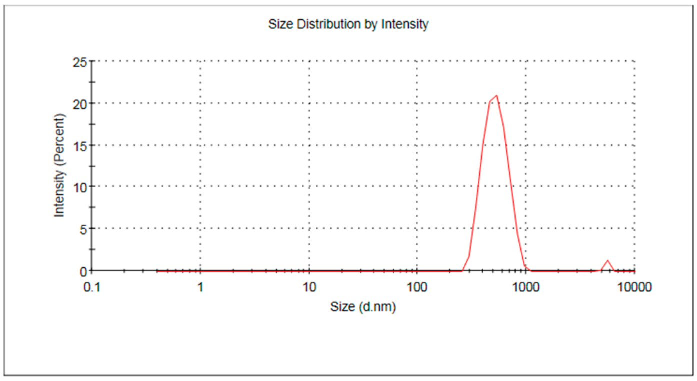

Z-Average (d.nm):	442.3
PdI:	0.798
Intercept:	0.999
Result quality :	Refer to quality report

	Size (d.nm):	% Intensity:	St Dev (d.n...
Peak 1:	179.6	84.4	23.64
Peak 2:	16.55	15.6	2.393
Peak 3:	0.000	0.0	0.000

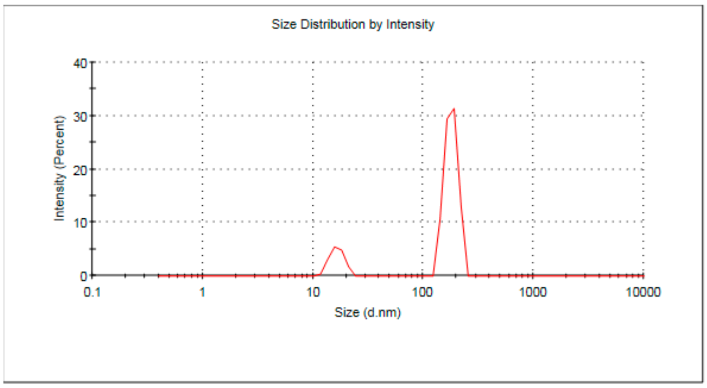

Figure S1. Malvern's Zetasizer graphs showing multiple measurements of particle size of CXB-GLR-PVP.

			Mean (mV)	Area (%)	St Dev (mV)
Zeta Potential (mV):	-22.3	Peak 1:	-22.3	100.0	7.50
Zeta Deviation (mV):	7.50	Peak 2:	0.00	0.0	0.00
Conductivity (mS/cm):	0.0170	Peak 3:	0.00	0.0	0.00
Result quality :	Good				

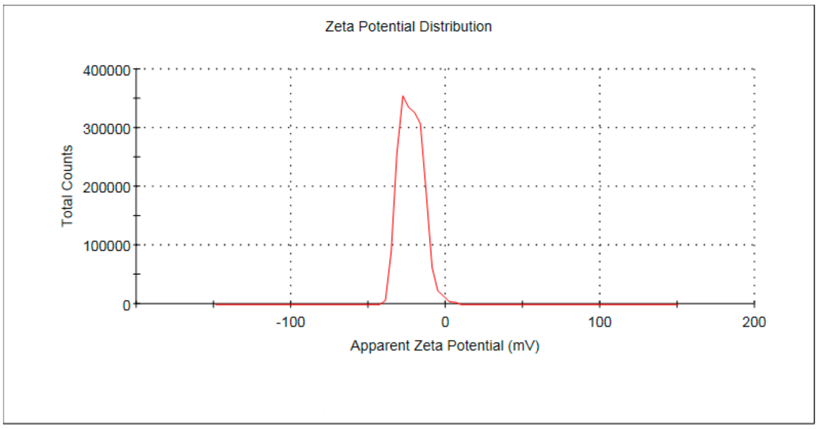

			Mean (mV)	Area (%)	St Dev (mV)
Zeta Potential (mV):	-20.3	Peak 1:	-20.3	100.0	4.00
Zeta Deviation (mV):	4.00	Peak 2:	0.00	0.0	0.00
Conductivity (mS/cm):	0.0168	Peak 3:	0.00	0.0	0.00
Result quality :	Good				

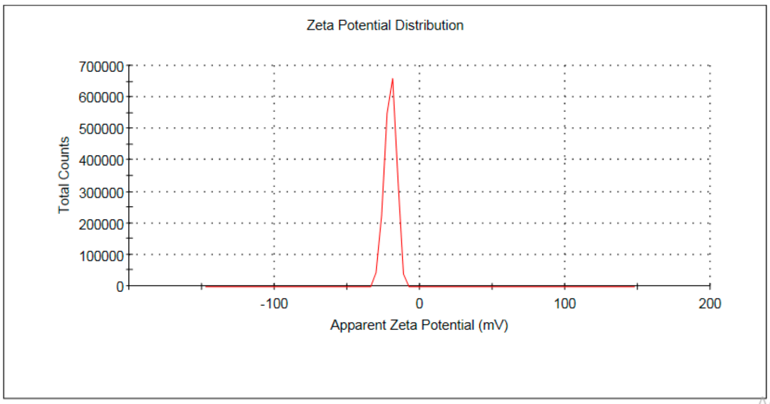

			Mean (mV)	Area (%)	St Dev (mV)
Zeta Potential (mV):	-20.3	Peak 1:	-20.3	100.0	4.00
Zeta Deviation (mV):	4.00	Peak 2:	0.00	0.0	0.00
Conductivity (mS/cm):	0.0168	Peak 3:	0.00	0.0	0.00
Result quality :	Good				

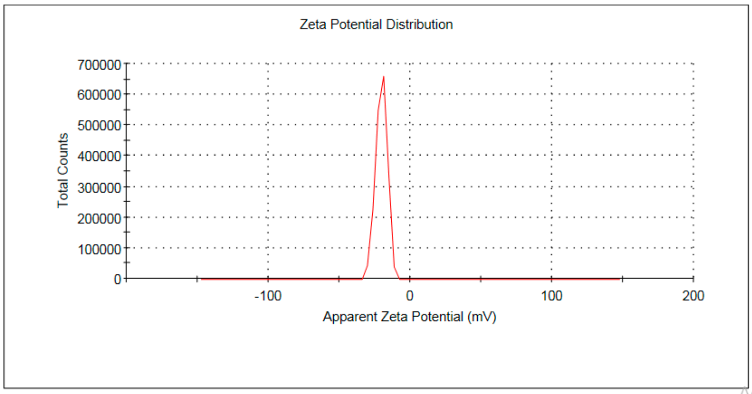

Figure S2. Malvern's Zetasizer graphs showing multiple measurements of ζ-potential of CXB-GLR-PVP.
